# Homogeneity of the coronary microcirculation in angina with non-obstructive coronary artery disease

**DOI:** 10.1093/ehjci/jeaf101

**Published:** 2025-03-24

**Authors:** Masahiro Hoshino, Roel Hoek, Ruurt A Jukema, Jorge Dahdal, Pepijn van Diemen, Pieter Raijmakers, Roel Driessen, Jos Twisk, Ibrahim Danad, Tsunekazu Kakuta, Juhani Knuuti, Paul Knaapen

**Affiliations:** Departments of Cardiology, Amsterdam UMC, Vrije Universiteit Amsterdam, De Boelelaan 1117, 1081 HV Amsterdam, The Netherlands; Departments of Cardiology, Amsterdam UMC, Vrije Universiteit Amsterdam, De Boelelaan 1117, 1081 HV Amsterdam, The Netherlands; Departments of Cardiology, Amsterdam UMC, Vrije Universiteit Amsterdam, De Boelelaan 1117, 1081 HV Amsterdam, The Netherlands; Departments of Cardiology, Amsterdam UMC, Vrije Universiteit Amsterdam, De Boelelaan 1117, 1081 HV Amsterdam, The Netherlands; Departamento de Enfermedades Cardiovasculares, Clinica Alemana de Santiago, Facultad de Medicina, Clinica Alemana Universidad del Desarrollo, Santiago, Chile; Departments of Cardiology, Amsterdam UMC, Vrije Universiteit Amsterdam, De Boelelaan 1117, 1081 HV Amsterdam, The Netherlands; Radiology, Nuclear Medicine and PET Research, Amsterdam UMC, Vrije Universiteit Amsterdam, Amsterdam, The Netherlands; Departments of Cardiology, Amsterdam UMC, Vrije Universiteit Amsterdam, De Boelelaan 1117, 1081 HV Amsterdam, The Netherlands; Epidemiology and Data Science, Amsterdam UMC, Vrije Universiteit Amsterdam, Amsterdam, The Netherlands; Departments of Cardiology, Amsterdam UMC, Vrije Universiteit Amsterdam, De Boelelaan 1117, 1081 HV Amsterdam, The Netherlands; Department of Cardiology, University Medical Center Utrecht, Heidelberglaan 100, 3584 CX Utrecht, The Netherlands; Department of Cardiology, Tsuchiura Kyodo General Hospital, Ibaraki, Japan; Turku PET Centre, Turku University Hospital and University of Turku, Turku 20520, Finland; Clinical Physiology, Nuclear Medicine and PET, Turku University Hospital and University of Turku, Turku 20520, Finland; Departments of Cardiology, Amsterdam UMC, Vrije Universiteit Amsterdam, De Boelelaan 1117, 1081 HV Amsterdam, The Netherlands

**Keywords:** microvascular resistance reserve, fractional flow reserve, [^15^O]H_2_O PET, coronary flow reserve, non-obstructive coronary artery disease

## Abstract

**Aims:**

The homogeneity of coronary microvascular dysfunction (CMD) across different myocardial territories in angina with non-obstructive coronary artery disease (ANOCA) patients is scarcely explored. This study investigates the variability in microvascular resistance reserve (MRR) across the 3 main perfusion territories of the coronary circulation to investigate the homogeneity or dishomogeneity of microcirculatory function.

**Methods and results:**

This *post hoc* analysis of the PACIFIC trials included symptomatic ANOCA patients with [^15^O]H_2_O positron emission tomography (PET) and three-vessel invasive fractional flow reserve (FFR). MRR was computed in the three main coronary branches by integrating PET-derived coronary flow reserve and invasive FFR. A total of 155 patients (50% male, age 59 ± 10 years) and 465 vessels (MRR: 3.92 ± 1.21) were included. There were no significant differences in MRR among the three coronary branches. Correlations in MRR among the three coronary branches were good (*r* = 0.76 to 0.86). The mean difference between MRR measurements in different arteries was small (2.4 to 7.5%), without any consistent directional bias. The overall intra-class correlation coefficient for absolute agreement was 0.80 (95% CI: 0.74–0.85), indicating good single-measure reliability. Approximately 80% (123/155) of patients showed diagnostic concordance of CMD (MRR ≤3.0) across the three vessels.

**Conclusion:**

In most ANOCA patients, microvascular function is homogeneously distributed across the three major coronary territories. Single-artery testing may suffice in many cases, aligning with guidelines. However, some patients exhibit notable inter-territorial variation, suggesting that multi-vessel evaluation may be prudent in borderline scenarios.

## Graphical Abstract

**Graphical Abstract:**
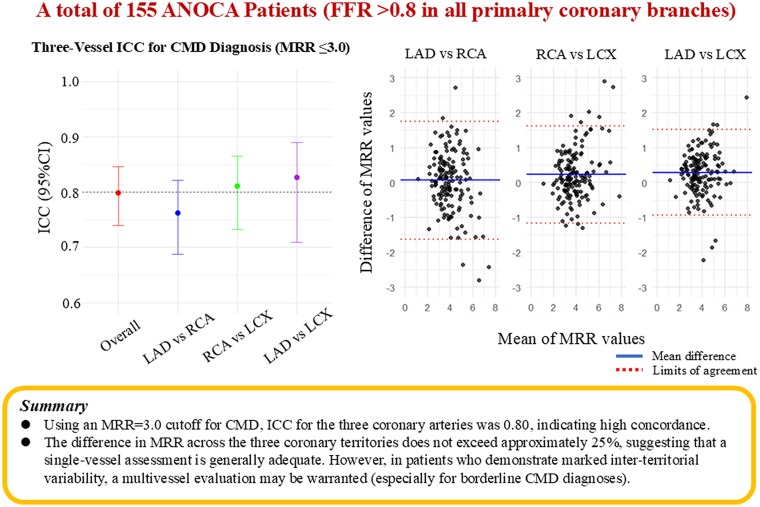

**See the editorial comment for this article ‘Homogeneous reduction of 15O-water and PET-determined myocardial flow reserve as a reflection of coronary microvascular dysfunction?’, by T.H. Schindler *et al*., https://doi.org/10.1093/ehjci/jeaf122.**

## Introduction

The importance of coronary microcirculation as a cause of angina pectoris and myocardial ischaemia has been well recognized, even without noticeable epicardial disease (ANOCA; angina with no obstructive coronary arteries). Consequently, testing protocols have been developed and endorsed by guidelines^1^ to detect coronary microvascular dysfunction (CMD). Mainly for practical reasons, these testing protocols generally recommend testing in one coronary artery, mostly the left anterior descending artery (LAD) artery. Such protocols implicitly presume then that CMD—if present—is homogeneously distributed within the myocardium of ANOCA patients. Nevertheless, although the homogeneous distribution of microcirculatory function is very plausible in healthy individuals, such a hypothesis is not *a priori* true in ANOCA patients. Therefore, it is unclear whether measurement in a single vessel is sufficient, and this uncertainty forms the central focus of the present study.

In some previous studies using bolus thermodilution-derived index of microcirculatory resistance (IMR) or coronary flow reserve (CFR), this hypothesis was even questioned.^2,3^ However, the methodology of Doppler or bolus thermodilution derived CFR and IMR is somewhat imprecise, with limited reproducibility and operator dependence.^4,5^ In addition, only a surrogate measure of microvascular (dys)function is obtained such as flow velocity or transit time (Tmn). For assessment of microvascular function, both techniques are confounded by either diffuse, non-obstructive, or hidden epicardial disease, which is often present to some degree even without visible obstructions.^6^ Absolute hyperemic flow and resting microvascular resistance depend on epicardial disease.^5^ To avoid such dependency on epicardial disease and mass, the index microvascular resistance reserve (MRR) was introduced in 2021.^7^ MRR, as defined by De Bruyne *et al*., provides a more reliable exploration of variations in microcirculatory function across the 3 main coronary artery territories.

In the initial studies, MRR was calculated during invasive coronary function testing by thermodilution and continuous infusion of a low rate of saline. It turned out to be an accurate methodology for characterizing the microcirculation, irrespective of epicardial disease and mass.^8^ In the present approach, we use a hybrid methodology for MRR assessment, based upon flow measurement by non-invasive positron emission tomography (PET) and invasively measured FFR in all three coronary branches.

This study aims to evaluate the homogeneity of MRR in ANOCA patients to assess the accuracy and relevance of current guidelines. By analysing the observed variations, we seek to assess whether they fall within the acceptable range of measurement error or biological variability.

## Methods

### Patient selection

This study is a *post hoc* analysis of the comparison of coronary CT angiography, SPECT, PET, and hybrid imaging for diagnosis of ischaemic heart disease determined by fractional flow reserve (FFR; PACIFIC 1) and Functional stress imaging to predict abnormal coronary FFR (PACIFIC 2) studies.^9,10^ Both studies were prospective, single centre, head-to-head comparative studies from 2012 to 2020 at Amsterdam University Medical Centers, location VU Medical Center in Amsterdam, The Netherlands. Patients with suspected obstructive CAD completed a two-week protocol including [^15^O]H_2_O PET prior to invasive coronary angiography (ICA) coupled with routine 3 vessel invasive FFR examination. Patients with FFR > 0.8 in all three primary coronary artery branches were defined as ANOCA. The current study included all patients with ANOCA in whom MRR could be calculated in all three primary coronary branches. The VU medical center institutional ethics review committee approved the study protocols and complied with the Declaration of Helsinki, with written informed consent obtained from 155 participants before study inclusion.

### PET procedure

PET scans were performed on a hybrid PET/CT equipment (Philips Gemini TF 64 or Ingenuity TF 128, Philips Healthcare, Best, The Netherlands). Medication was neither discontinued nor changed during the execution of the study protocol. The scan procedure involved a dynamic 6-min scanning protocol that initiated simultaneously with an injection of 370 MBq [^15^O]H_2_O during resting and adenosine-induced hyperemic conditions (140 µg/kg/min). Intravenous adenosine was administered to induce hyperemia, initiated 2 min before the stress scan for maximal vasodilation. The participants were asked to abstain from caffeine or xanthine intake 24 h before the PET scan. Parametric segmental myocardial blood flow (MBF) values were allocated to their corresponding vascular territory according to the American Heart Association 17-segment model after correction for coronary dominance based on information obtained from ICA.^11^

Images were analysed using the in-house developed CardiacVUer software (Amsterdam UMC, Vrije Universiteit Amsterdam, The Netherlands).^12^ This software was utilized for sub-analyses in which regional hyperemic MBF (hMBF) and resting MBF (rMBF) were examined. MBF was defined as the mean MBF of the entire vascular territory. CFR was defined as the ratio of hMBF to rMBF.^13^

### ICA and physiological assessments

ICA was performed according to standard clinical protocols.^9^ Patients were instructed to refrain from the intake of xanthine or caffeine 24 h prior to ICA. All major coronary arteries (>2 mm) were routinely interrogated by FFR, irrespective of stenosis severity and imaging results. To induce maximal coronary hyperemia, adenosine was administered either intracoronarily (150 µg) or intravenously (140 mg/kg/min). FFR was calculated as the ratio of mean distal intracoronary to aortic guiding pressure during hyperemia.

MRR was derived based on the theoretical framework by the De Bruyne *et al*. by combining invasive FFR measurements and non-invasive PET flow measurements.^7^ The formula used in the current analysis is a quotient of CFR and FFR with the correction for the impact of haemodynamics, as follows:


$$\mathrm{MRR}=(\mathrm{CFR}/\mathrm{FFR})\times (P_{a,\;\mathrm{rest}}/P_{a,\;\mathrm{hyper}})$$

CFR indicates PET-derived CFR, FFR indicates pressure-wire derived FFR, and *P_a_*_, rest_ and *P_a_*_, hyper_ indicate mean aortic pressure during non-hyperemic and maximal hyperemic PET, respectively.

Based on prior research,^14,15^ we adopt a prognostically significant threshold of MRR ≤ 3.0 to define CMD.

### Statistical analysis

Continuous variables were presented as mean ± SD or median with IQR, based on their distribution. Categorical variables were expressed as frequencies and percentages. We applied the Friedman test for FFR and repeated measures ANOVA for MRR and CFR to assess differences among the three coronary vessel: LAD, right coronary artery (RCA), and left circumflex artery (LCX). Pearson correlation was used for pairwise comparisons of MRR values between each of the three coronary branches: LAD and RCA, RCA and LCX, LAD and LCX. The intra-class correlation coefficient (ICC) was used to assess the absolute agreement of MRR measurements across the three coronary arteries. A two-way mixed-effects model was employed to evaluate the measurements’ homogeneity for agreement. ICC values were calculated for the overall set of coronary arteries as well as for each pair of arteries (LAD and RCA, RCA and LCX, LAD and LCX) to evaluate the agreement of the measurements. The Bland–Altman analysis was employed to assess the agreement between MRR measurements across the three coronary arteries. To evaluate the variability of MRR for each coronary artery branch (LAD, RCA, and LCX), we calculated the coefficient of variation (CV). Previous reports on regional MBF and FFR found reproducibility of around 50% and 3%, respectively.^16,17^ Considering these values and a total sample size of 155 produces a minimal detectable difference of ∼25% at 80% power and *P* < 0.05.

The concordance rate between the 3 territories was defined as the percentage of patients whose MRR values across all three coronary arteries were either all below 3.0 or all above 3.0. We performed univariable linear regression using the three-vessel means MRR as the dependent variable to assess baseline predictors. A multi-variable model was then constructed for factors showing univariable significance (*P* < 0.10).

Statistical significance was determined by a two-sided *P* value <0.05 for all analyses. All statistical computations were executed using R version 4.4.3. (R Foundation for Statistical Computing, Vienna, Austria).

## Results

**Figure 1:**
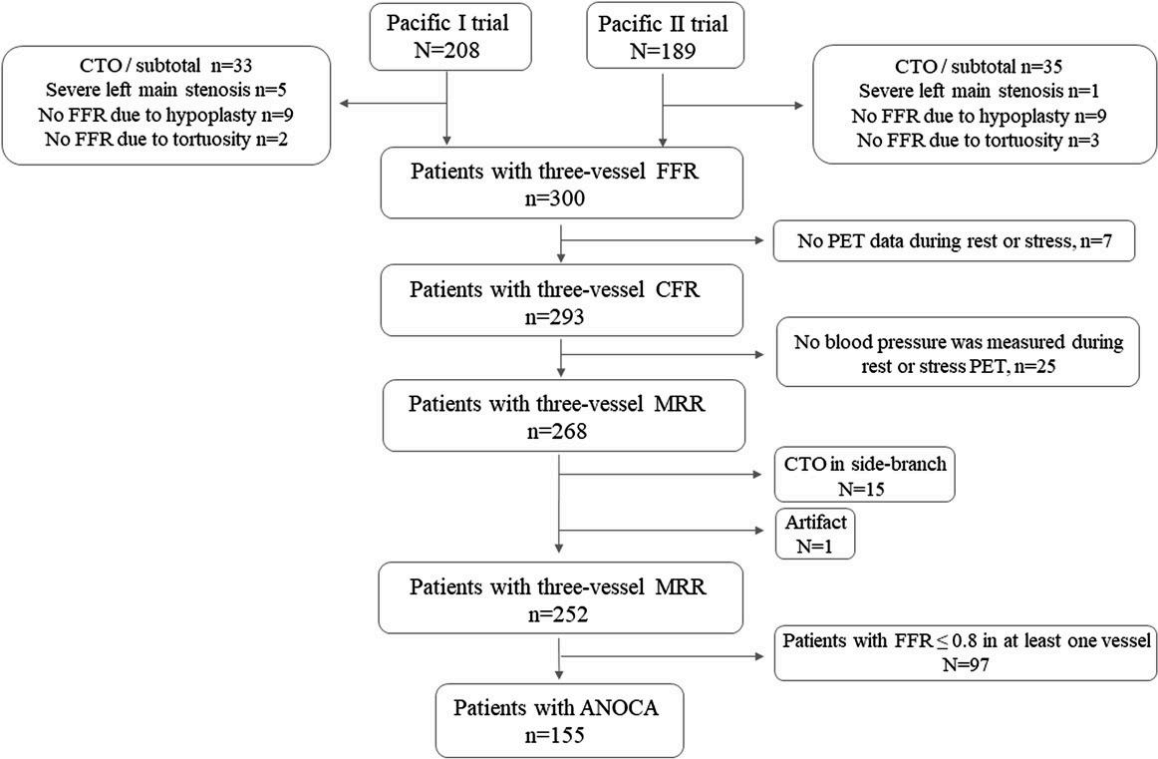
Study flow chart. Flow chart of participants from PACIFIC 1 trial and PACIFIC 2 trial at Amsterdam UMC. ANOCA, angina with non-obstructive coronary artery disease; CTO, chronic total occlusion; CFR, coronary flow reserve; FFR, fractional flow reserve; MRR, microvascular resistance reserve; PET, positron emission tomography.

*Figure 1* shows a detailed flow chart on physiology indices. Chronic total occlusion (CTO), severe left main stenosis, and a lack of FFR measurement excluded patients from the study population. Of the 397 patients in the PACIFIC 1 and 2 studies, 268 (68%) patients had three-vessel MRR measurements. Sixteen patients were excluded due to the presence of CTO in a side branch (*n* = 15) or an artefact (*n* = 1). Of these, 97 patients had at least one vessel with an obstructive stenosis (FFR ≤ 0.8). As a result, the final analysis included 155 patients with ANOCA.

**Figure 2:**
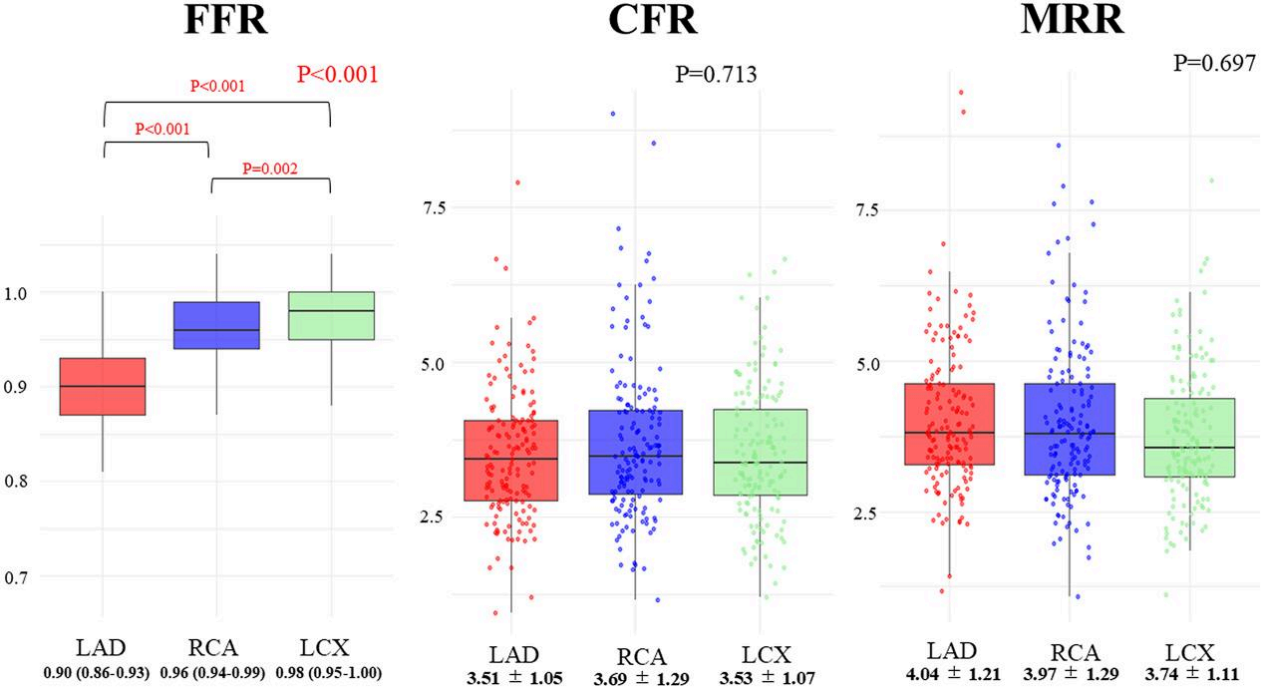
Comparative analysis of FFR, CFR, and MRR across coronary branches. The comparison of FFR, CFR, and MRR across LAD, RCA, and LCX arteries. ANOCA, angina with non-obstructive coronary artery disease; CTO, chronic total occlusion; CFR, coronary flow reserve; FFR, fractional flow reserve; MRR, microvascular resistance reserve; PET, positron emission tomography; LAD, left anterior descending artery; RCA, right coronary artery; LCX, left circumflex artery.

Patient characteristics are described in *Table 1*. In short, mean age was 59.0 ± 9.6 years, and 78 (50%) were male. A total of 24 patients (16%) had a history of myocardial infarction. Lesion characteristics are described in *Table 2*: In the overall cohort, FFR was slightly lower in the LAD region than in other areas (FFR: LAD 0.90 (0.86–0.93), RCA 0.96 (0.94–0.99), LCX 0.98 (0.95–1.00), and *P* < 0.001). There was no significant difference in CFR among the regions (CFR: LAD 3.51 ± 1.05, RCA 3.69 ± 1.29, LCX 3.53 ± 1.07, and *P* = 0.713), and similarly, there were no significant differences in MRR among the three coronary branches (MRR: LAD 4.04 ± 1.21, RCA 3.97 ± 1.29, LCX 3.74 ± 1.11, and *P* = 0.697) (*Figure 2*). FFR, CFR, and MRR in patients with an epicardial lesion (FFR ≤ 0.80) are also presented in Supplementary data online, *Table S1*. CV for MRR measurements were 30.0% for LAD, 32.6% for RCA, and 29.5% for LCX. There was no correlation between FFR and MRR within the same coronary territories for the LAD (*r* = 0.05, *P* = 0.546), RCA (*r* = −0.03, *P* = 0.740), or LCX (*r* = −0.03, *P* = 0.756).

**Table 1:** Baseline characteristics

	All patients n = 155
Characteristics	
Male	78 (50.3%)
Age, years	59.0 ± 9.6
BMI, kg/m^2^	27.3 ± 4.0
Prior PCI	56 (30.1%)
Prior MI	24 (15.5%)
Cardiovascular risk factors	
Diabetes mellitus	24 (15.5%)
Hypertension	85 (54.8%)
Hypercholesterolemia	65 (41.9%)
Currently smoking	26 (16.8%)
Family history of CAD	84 (54.2%)
Medication	
Antiplatelet therapy	138 (89.0%)
β-blocker	93 (60.0%)
Calcium channel blocker	43 (27.7%)
ACE-inhibitor	44 (28.4%)
ARB	23 (14.8%)
Statin	121 (78.1%)
Long-acting nitrate	29 (18.7%)

BMI, body mass index; PCI, percutaneous coronary intervention; MI, myocardial infarction; CAD, coronary artery disease; ACE, angiotensin-converting enzyme; ARB, angiotensin II receptor blockers.

**Table 2:** Vessel characteristics

	All vessels *N* = 155 (465 vessels)				
	Overall	LAD	RCA	LCX	*P* value
FFR	0.95 (0.90–0.99)	0.90 (0.86–0.93)	0.96 (0.94–0.99)	0.98 (0.95–1.00)	<0.001
CFR	3.58 ± 1.15	3.51 ± 1.05	3.69 ± 1.29	3.53 ± 1.07	0.713
MRR	3.92 ± 1.21	4.04 ± 1.21	3.97 ± 1.29	3.74 ± 1.11	0.697

FFR, fractional flow reserve; CFR, coronary flow reserve; MRR, microvascular resistance reserve.

### Correlation and variability in MRR

**Figure 3:**
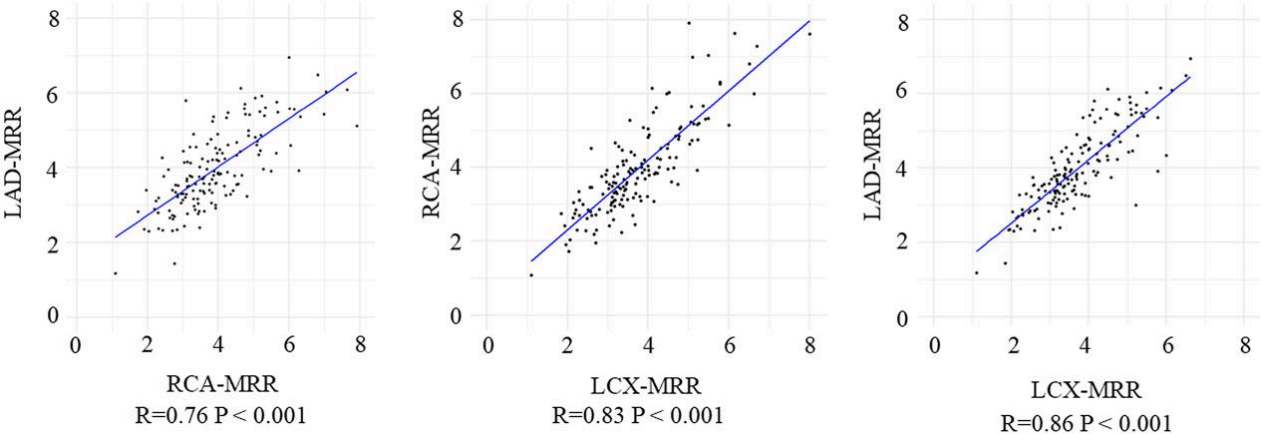
Correlation between each branch in the three-vessel MRR. ANOCA, angina with non-obstructive coronary artery disease; CTO, chronic total occlusion; CFR, coronary flow reserve; FFR, fractional flow reserve; MRR, microvascular resistance reserve; PET, positron emission tomography; LAD, left anterior descending artery; RCA, right coronary artery; LCX, left circumflex artery.

**Figure 4:**
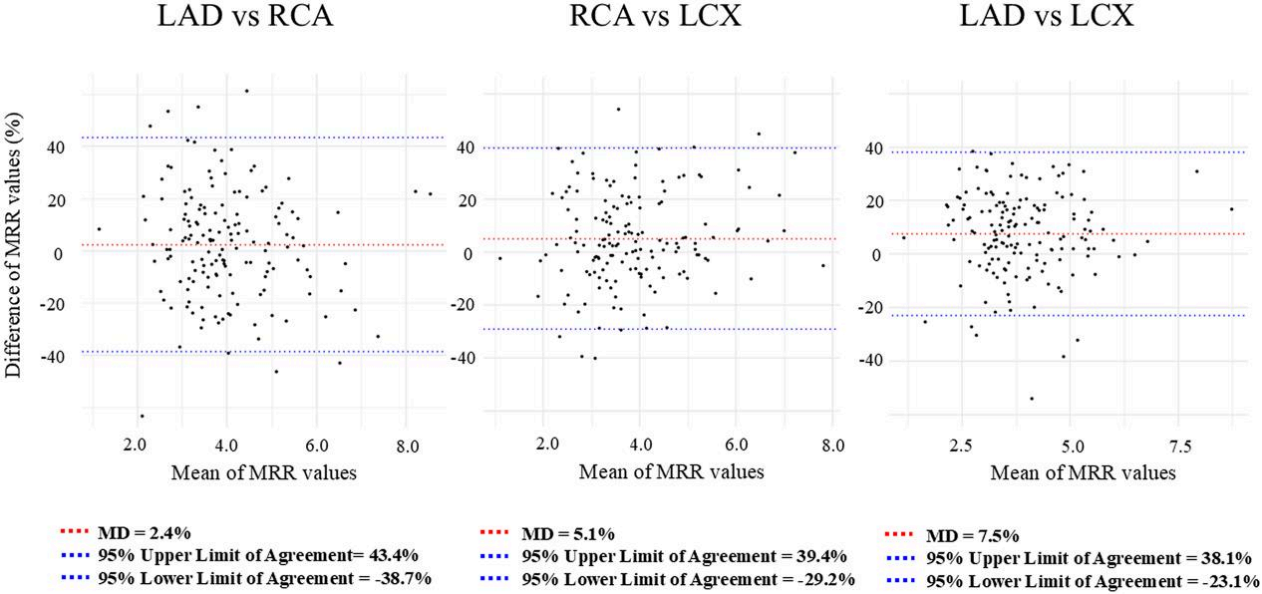
Bland-Altman plot across three-vessel MRR. Bland–Altman plots for MRR across three coronary vessels: LAD/RCA, LAD/LCX, and RCA/LCX. Each plots shows the mean difference (MD) between the two measurements at the center, with upper and lower limits of agreement defined as MD ± 1.96 times the standard deviation of the differences. ANOCA, angina with non-obstructive coronary artery disease; CTO, chronic total occlusion; CFR, coronary flow reserve; FFR, fractional flow reserve; MRR, microvascular resistance reserve; PET, positron emission tomography; LAD, left anterior descending artery; RCA, right coronary artery; LCX, left circumflex artery.

The correlation coefficients for MRR between vessels were *r* = 0.76 (0.69–0.82) for LAD-RCA, *r* = 0.83 (0.78–0.88) for RCA-LCX, and *r* = 0.86 (0.81–0.89) for LAD-LCX. (*Figure 3*) The overall ICC was 0.80 (95% CI: 0.74–0.85) for absolute agreement, indicating good single-measure concordance (*F* = 13.8, *P* < 0.001). Pairwise analysis showed: (i) LAD and RCA: ICC = 0.76 (95% CI: 0.69–0.82); (ii) RCA and LCX: ICC = 0.83 (95% CI: 0.78–0.88); (iii) LAD and LCX: ICC = 0.86 (95% CI: 0.81–0.89) (Central illustration). The Bland-Altman analysis indicated MRR variability across coronary branches. Mean differences were 2.4% (Lower limit: −38.7 to upper limit: 43.4) for LAD/RCA, 5.1% (−29.2 to 39.4) for RCA/LCX, and 7.5% (−23.1 to 38.1) for LAD/LCX (*Figure 4*).

### CMD prevalence and territorial congruence

Concordance of MRR across the three major territories was found in 79% (123/155) of the patients when using the strict binary cut-off criterium of MRR = 3.0. We found no significant differences when comparing CMD prevalence across coronary branches for MRR 3.0 (LAD 16%, RCA 20%, LCX 22%, *P* = 0.140). CMD prevalence varied across individual vessels: When using the binary cut-off criterium, the majority (70%) had no CMD, 12% had 1-vessel CMD, 8% had 2-vessel CMD, and 10% had 3-vessel CMD, respectively. We observed a 21% (32 out of 155 cases) discongruence in CMD diagnosis among the 3 major branches. However, many discordances occurred near the cut-off-value of 3.0.

### Predictors of lower three-vessel mean MRR

Univariable analysis showed only prior MI was significantly associated with lower three-vessel mean MRR (*P* = 0.007), while diabetes and current smoking showed borderline significance (*P* < 0.10). In the multi-variable model, prior MI remained independently predictive of lower three-vessel mean MRR (*P* = 0.008) (*Table 3*)

**Table 3:** Univariable and multi-variable linear regression analyses of the three-vessel mean MRR

	Univariable analysis		Multi-variable analysis 1	
	β	*P* value	β	*P* value
Age	−0.008	0.385		
Male	−0.017	0.347		
BMI	−0.019	0.410		
Diabetes mellitus	−0.439	0.080	−0.345	0.160
Hypercholesterolemia	−0.138	0.454		
Hypertension	−0.226	0.215		
Current smoker	−0.406	0.094	−0.421	0.075
Prior myocardial infarction	−0.675	0.007	−0.658	0.008

BMI, body mass index; MRR, microvascular resistance reserve.

## Discussion

This study elucidates the variability of microvascular function across different coronary territories in patients with ANOCA. By employing a comprehensive evaluation using [^15^O]H_2_O PET and invasive FFR measurements, our findings revealed good correlations of MRR across the three primary coronary branches. The difference in MRR among the three major coronary branches generally does not exceed 25%, and the overall ICC for absolute agreement was 0.8—together indicating high concordance across the vessels. Thus, it can be generally considered that differences in MRR among the three vessels fall within the range of measurement error and biological variability associated with the hybrid methodology used. As such, it can be hypothesized that microcirculatory (dys)function is rather a homogeneously distributed process across the three main territories in the majority of ANOCA patients, suggesting that coronary function testing in a single artery, typically the LAD, may suffice as an index artery. Nonetheless, marked inter-territorial variability in MRR is observed in a non-negligible proportion of patients, suggesting that a multi-vessel evaluation may be warranted for these patients to ensure a more comprehensive assessment of coronary microvascular (dys)function.

### Heterogeneity of microvascular dysfunction

Previous studies have reported that the frequency of CMD among the three coronary arteries does not completely coincide in about one-third of the cases to one-half of the cases.^3,18^ However, these studies defined CMD based on CFR and IMR criteria, which are not specific to the microcirculation and may be confounded by hidden or overt epicardial disease. In addition, the measurement variability of bolus thermodilution derived IMR or CFR is larger than for MRR (if measured invasively) and at least as large as for MRR in our hybrid approach.^5^ Since CFR and IMR are influenced by epicardial disease,^3^ it is challenging to accurately evaluate ANOCA patients if the issue of epicardial cardiovascular disease has not been excluded or at least quantified using FFR. Many previous studies on ANOCA have defined the absence of obstructive CAD by performing diagnostic ICA to confirm that visual stenosis is less than 50%,^18,19^ which may be accompanied by FFR-values as low as 0.70.^20^ There have been very few clinical studies where ANOCA populations were defined solely based on negative FFR and even then, negative has a range of 0.81–1.00, which means that without FFR correction (as done in MRR), errors of up to 20% in addition to measuring variability, may occur.^20^ Furthermore, IMR has various limitations when measuring microcirculation, which might emphasize differences among the coronary arteries. For instance, Tmn is a surrogate marker of MBF and shows variability in measurements between procedures.^4^ IMR is significantly influenced by the subtended mass, as well as anatomical factors such as vessel diameter and length.^21,22^ Since these anatomical factors vary considerably among the three coronary arteries, comparing IMR across the three coronary arteries complicates the accurate assessment of microvascular dysfunction. Additionally, given the lower reproducibility of IMR compared to MRR,^5^ differences in measurement timing for each artery could further influence the results. Considering these limitations, it remains uncertain whether previous studies accurately reflect differences in CMD among the coronary regions.

### Advancements in CMD assessment through PET-derived blood flow measurements

In contrast, our study evaluated the microcirculation of the all three major coronary arteries by using MRR measured by PET-derived CFR combined with invasively measured FFR and using the blood pressure data simultaneously measured with the PET flow measurements. [^15^O]H_2_O PET is recognized as the non-invasive gold standard for quantifying absolute MBF.^23,24^ The high reproducibility of invasively measured FFR (<3%^17^) enables a fair and reliable comparison of MRR across the three coronary branches, even when combining invasive wire-based FFR measurements and PET-derived CFR at different timings. This consistency supports the reliability of MRR as a diagnostic tool in evaluating coronary microcirculation across different branches, reducing concerns about significant methodological variability.

ICC results indicated low variability in MRR measurements across the three coronary arteries. Furthermore, mean differences in MRR for the three coronary branches are within 10%, with CVs also within ∼30%. These findings suggest that MRR measurements are relatively uniform across the coronary arteries, reflecting minimal inter-vessel variability, especially considering the inevitable presence of at least some measurement error and biological variability. Since MRR measured by PET-derived CFR and invasive FFR is independent of myocardial mass and anatomical factors, it serves as an optimal parameter for evaluating differences in microcirculation among coronary arteries.^7^ This approach advances our understanding of the dynamics of pure coronary microcirculation across different branches in a way not seen in previous studies.^3,18^ In a previous study evaluating MBF reproducibility in healthy subjects using [^15^O]H_2_O PET, regional reproducibility was found to be worse than overall reproducibility.^16^ While changes in response to adenosine may explain some of the variability, a certain amount of measurement error is also likely. Thus, in our study’s assessment of regional variability, part of the observed variation may indeed be attributable to the measurement’s inherent error.

## Future directions

It is unlikely that [^15^O]H₂O PET will be performed in every patient as part of routine clinical care. Instead, it serves as a gold standard reference to clarify who might need additional microvascular measurements beyond standard diagnostic protocols. In the future, combining PET-based flow measurements with FFRct could possibly enable a fully non-invasive evaluation of both obstructive CAD and CMD, further enhancing the comprehensive detection of coronary pathophysiology.

## Limitation

This study has several limitations that should be acknowledged. First, the retrospective nature of the study introduces potential biases in patient selection (*Figure 1*). Secondly, patients in this study had an incomplete microcirculatory evaluation protocol due to not assessing spasm, requiring endothelial function tests with Acetylcholine. Although distinguishing between impaired microvascular conductance and spasmogenic dysregulation is key for effective symptom management in patients with ANOCA, this was beyond our goal of studying homogeneity of the microcirculation.^25^ Thirdly, it would be ideal in our study to have comparative data from truly healthy, normal individuals to test the homogeneity of the microcirculation in a healthy population. Although that is highly likely, such study is prohibited by the Helsinki rules. Fourthly, it is important to note that MRR cut-offs have been proposed in previous reports, all of which were measured using invasive wire techniques,^14,15^ differing from our hybrid approach that utilizes PET-derived CFR flow. Fifth, we excluded patients with FFR ≤ 0.80, in whom territory-wide hyperemic MBF may overestimate myocardial flow if epicardial disease is present. Such cases require a different analytic framework (e.g. segmental or defect-based MBF) for accurate MRR calculations.^26^ Consequently, our findings apply mainly to ANOCA patients (FFR > 0.80). Sixth, our diagnosis of ANOCA depended primarily on the presence of angina-like symptoms and the absence of obstructive coronary disease (FFR > 0.80). We did not conduct systematic evaluations to exclude non-cardiac causes of chest pain nor specific testing to exclude pericarditis or myocarditis as causes of chest pain. Seventh, we did not perform [^18^F]FDG scans to identify or exclude infarcted segments, relying solely on clinical history to determine prior myocardial infarction. In addition, we did not exclude the 24 patients with prior infarctions, which could complicate the interpretation of microvascular function, given that vasodilatory responses in scarred myocardium are often diminished.^27^ Lastly, this study did not obtain absolute coronary flow and resistance values, which are typically acquired through direct invasive measurement of MRR. Instead, we highlight a possible alternative method for calculating MRR. Based on the current results, however, and considering the challenges of implementing this method in clinical practice, our findings suggest that invasively measured MRR may be sufficient for single-vessel analysis.

## Conclusion

Our findings indicate that microvascular function in most ANOCA patients is homogeneously distributed across the three major coronary arteries, with MRR differences typically under 25% and an ICC of 0.8, indicating high concordance. Consequently, single-artery coronary function testing—commonly the LAD—appears sufficient in many cases, aligning with current guidelines. However, a non-negligible subset exhibited greater inter-territorial variation. If an MRR result is near the diagnostic threshold, even small shifts could alter classification, making multi-vessel evaluation prudent for borderline or complex scenarios. Further research is warranted to refine patient selection for either single- or multi-vessel assessment.

## Supplementary Material

jeaf101_Supplementary_Data

## Data Availability

The data that support the findings of this study are available from the corresponding author upon reasonable request. Such requests are subject to approval by the responsible authority at Amsterdam UMC. All requests will be reviewed on a case-by-case basis in accordance with applicable ethical and privacy guidelines.
