# Supplementary material for: Homogeneity of the coronary microcirculation in angina with non-obstructive coronary artery disease
Source: Eur Heart J Cardiovasc Imaging. 2025 Mar 24;26(7):1120–7. doi: 10.1093/ehjci/jeaf101 (PMC12206580; doi:10.1093/ehjci/jeaf101)
Supplement: jeaf101_Supplementary_Data [file jeaf101_supplementary_data.docx]

**Supplemental Table 1. Vessel characteristics in patients with  FFR ≤ 0.80**

|  | **N=97 (291 vessels)** | | | |
| --- | --- | --- | --- | --- |
|  | Overall | LAD | RCA | LCX |
| FFR | 0.84 (0.75-0.93) | 0.75 (0.64-0.79) | 0.91 (0.84-0.95) | 0.91 (0.81-0.96) |
| CFR | 2.81 ± 0.93 | 2.70 ± 0.91 | 2.92 ± 0.99 | 2.83 ± 0.88 |
| MRR | 3.63 ± 1.16 | 4.02 ± 1.21 | 3.48 ± 1.16 | 3.38 ± 1.00 |

Abbreviations: FFR, fractional flow reserve; CFR, coronary flow reserve; MRR, microvascular resistance reserve.
